# Merkel Cell Carcinoma: Chemotherapy and Emerging New Therapeutic Options

**DOI:** 10.1155/2013/327150

**Published:** 2013-02-10

**Authors:** Laura Desch, Rainer Kunstfeld

**Affiliations:** Universitätsklinik für Dermatologie, AKH, Medizinische Universität Wien, Währinger Gürtel 18-20, 1090 Wien, Austria

## Abstract

Merkel cell carcinoma (MCC) is a rare neuroendocrine skin tumor that typically occurs in elderly, immunosuppressed patients. Infection with Merkel cell virus (MCV) and immunosuppression play an important role in the development of MCC. Different staging systems make it difficult to compare the existing clinical data. Furthermore, there predominantly exist single case reports and case series, but no randomized controlled trials. However, it is necessary to develop further therapy options because MCC tends to grow rapidly and metastasizes early. In the metastatic disease, therapeutic attempts were made with various chemotherapeutic combination regimens. Because of the high toxicity of these combinations, especially those established in SCLC, and regarding the unsatisfying results, the challenge is to balance the pros and cons of chemotherapy individually and carefully. Up to now, emerging new therapy options as molecular-targeted agents, for example, pazopanib, imatinib, or somatostatin analogues as well as immunologicals, for example, imiquimod and interferons, also showed less success concerning the disease-free response rates. According to the literature, neither chemotherapy nor molecular-targeted agents or immunotherapeutic strategies have shown promising effects in the therapy of the metastatic disease of MCC so far. There is a great demand for randomized controlled studies and a need for an MCC registry and multicenter clinical trials due to the tumors curiosity.

## 1. Clinical Features

Merkel cell carcinoma (MCC) of the skin, formerly called trabecular carcinoma, is a rare, highly malignant neuroendocrine tumor. Clinically only a presumptive diagnosis can be achieved. Clinical features that may serve as clues in the diagnosis of MCC are summarized in the acronym AEIOU: asymptomatic/lack of tenderness, expanding rapidly, immune suppression, older than age 50, and UV-exposed site on a person with fair skin [1]. The definitive diagnosis is made by histology and immunohistopathology depicting intermediate filaments and neuroendocrine markers [2]. The incidence of MCC has been rising in recent years [3, 4]. 5 years after diagnosis, overall survival was 40% and the age-adjusted and sex-adjusted survival was 54% [5].

Infection with the Merkel cell virus (MCV) and immunosuppression are the key factors in the development of MCC. The relative risk for MCC is about 13-fold higher in HIV [6] and about 5-fold higher in solid organ transplantation recipients [7] than in the general population. On the one hand, these features might also explain the increasing incidence of MCC. On the other hand, they might be key factors for the successful treatment of MCC.

The stage of patients with MCC has prognostic impact and influences the therapy offered. Over the years, different staging systems of MCC have been applied making it difficult to compare clinical data. In 2010, the American Joint Committee on Cancer (AJCC) Staging system first included the staging for MCC [8]. Duprat et al. [9] summarized AJCC Staging system and five-year survival data from Lemos et al. [5] in a table. Clinicians and researchers should be cautious when comparing clinical studies which applied older systems and with those which applied the recent AJCC Staging system.

MCC mostly appears as a hemispherical, firm-elastic, and reddish-livid tumor with a smooth surface. It is typically located on sun exposed areas like head, neck, extremities, and upper body [10]. Because of its harmless appearance, this rare neuroendocrine tumor, first described by Toker in 1972 [11], is often misdiagnosed, for example, as a cyst [1]. Interestingly, the clinically suspected diagnosis has only been made correctly for one percent of the cases [1, 12]. The MCC grows rapidly, is highly aggressive, and metastasizes early locoregionally as well as distantly [1, 13] illustrating the importance of comprehensive strategies to control disseminated diseases. 

## 2. Therapeutic Options

The rareness of the disease and, possibly also the comorbidities have contributed to the lack of prospective clinical studies evaluating the efficacy of therapeutic options. Up to now, only one randomized clinical study was published [34]. All other publications refer uncontrolled data from case series or even retrospective case descriptions.

The only randomized controlled clinical study published in the field of MCC compared regional adjuvant radiotherapy with observation [34]. While adjuvant radiotherapy significantly reduced the probability of regional recurrence, it had no effect on the overall survival. The authors wrote that the introduction of sentinel node dissection decreased the recruitment rate for this study. Surgery is considered the mainstay of treatment for MCC [10]. However, there is no evidence basis for this treatment option. A prospective evaluation of sentinel node dissection in MCC was described only in 9 patients [35].

## 3. Chemotherapeutic Options: Polychemotherapy and Monotherapeutic Options

Basically MCC is assumed to be a chemosensitive tumor [12, 36, 37] but to date no broadly accepted treatment algorithm exists. Supportively to primary excision, chemotherapy is used on MCC stages III (lymph node metastasis) and IV (distant metastases) after the AJCC Staging system. It may be applied either alone or in combination with radiotherapy. Furthermore, chemotherapeutics are used in locally advanced disease, as palliative measure or in case of recurrences [37].

The following data refers to retrospective case series or single case reports, except [14, 15] and (Table 1) [19], which are prospective case series. Several groups tried to treat MCC with chemotherapeutic regimens commonly used for small cell lung cancer (SCLC) because of histopathological and cytochemical similarities—besides both of them are considered as neuroendocrine tumors [16, 38–42]. These regimens combined carboplatin, cisplatin, and etoposide, cyclophosphamide with vincristine, doxorubicin, prednisone, bleomycin, or 5-fluorouracil (Table 1). They were reported to provide a good initial regression of the lesion, but recurrences occurred mainly within 4 to 15 months. All these patients had locoregional or distant metastases. These potential benefits should be weighed against the possible adverse reactions of these therapeutic attempts. Some of these rather old patients died from sepsis caused by chemotherapy-induced leukopenia [40], progressive renal failure [17], or impaired hepatic function [37]. 

In a retrospective analysis of a series of 251 patients from a single centre treated between 1970 and 2002 (Table 1) [24], the use of adjuvant chemotherapy was associated with decreased survival. 28 of 237 patients presenting with locally advanced disease or locoregional metastases received adjuvant chemotherapy and showed a 5-year disease-specific survival rate of 28% compared to 73% of 209 patients without receiving chemotherapy. Furthermore, 67 node-positive patients receiving chemotherapy were associated with a lower survival rate compared to not-receiving chemotherapy.

Beside polychemotherapy with burdensome toxicity, there are better tolerated monotherapeutic options like etoposide and anthracyclines [10, 25, 37]. Liposomal doxorubicin together with radiotherapy (*n* = 5) yielded rapid response, but showed less adverse reactions, for example, gastrointestinal disorders. Tumor proceeded within 1 to 3 months (Table 1) [25]. Orally administered etoposide achieved complete responses in 3 out of 4 patients (75%) and two of them were comparatively long lasting (16 and 36 months) (Table 1) [26]. 

There are no randomized studies that compare different chemotherapy regimens. In 204 patients, the most common regimens used were cyclophosphamide, doxorubicin (or epirubicin), vincristine plus/or minus prednisone, and etoposide combined with cisplatin (or carboplatin). A relevant difference in the response rate could not be described (Table 1) [27]. 

Although high remission rates were reported after chemotherapy (up to 70–75% [25, 37]), but no such prolongation of survival [10]. Intensity of chemotherapeutic therapy and response rates did not correlate [37]. Main therapy still is wide excision of the tumor with or without node dissection, often in combination with radiotherapy. In comparison, chemotherapy has been used rarely [45]. 

Up to now, the literature does not provide adequate, sufficient data to support the use of chemotherapy.

## 4. Molecular-Targeted Agents

Davids et al. (Table 2) [28] treated a patient who suffered from metastatic pulmonary lesions with pazopanib, which is a small-molecule tyrosine kinase inhibitor acting against vascular-endothelial-growth-factor-receptors- (VEGFR-) 1, 2, and 3 and against platelet-derived-growth-factor-receptor- (PDGFR-) *α* and *β*. The rationale was that MCCs have been shown having upregulated VEGFR [46, 47] and PDGFR [48, 49]. Pazopanib can be administered orally. It was well tolerated and provided partial response of the pulmonary lesions as well as complete response of the primary lesion, but disease recurred or rather progressed 4 months later. In this case, every other therapy that had been tried before (surgery, radiotherapy, etoposide and carboplatin, paclitaxel, tegafur, and 5-chloro-2,4-dihydroxypyridine and oxonic acid) also led to partial or complete response and lesions recurred 4–8 and once 24 months later. In this paper, the authors conclude that pazopanib appears to have a promising antitumor and antiangiogenic function and a good oral bioavailability; as per them, this has been demonstrated in preclinical studies. 

In this patient, single nucleotide polymorphism (SNP) in PDGFR-*α* gene was found, namely, 1432T>C mutation in codon 478. The same mutation was shown in two other samples of MCCs that were examined by this group and in three other patients with MCC from another study [49]. It is not clear whether this mutation leads to an activation of PDGFR-*α* and consequently to a better response to tyrosine kinase inhibitors, for example, pazopanib or not.

Similarly well tolerated is another tyrosine kinase inhibitor called imatinib mesylate (Gleevec) (Table 2) [29]. KIT (CD117), a tyrosine kinase receptor which belongs to the same family of tyrosine kinase receptors like PDGFR-*α*, is reported to be highly expressed (84–95%) in MCCs [50–52]. Activating mutations of KIT were found in 88,2% (*n* = 127) of gastrointestinal stromal tumors (GISTs) and showed a significantly higher partial response rate to imatinib for exon 11 KIT mutation (83.5%) compared to exon 9 KIT mutation (47.8%) or no found mutation (0.0%) [53]. Because of the successful treatment of KIT expressing GISTs [53, 54] and because of the recent insight that KIT receptor activation through its ligand stem cell factor (SCF) stimulates growing of MCC cells in vitro [55], imatinib was assumed to be a promising therapeutic option for MCC. In fact, there were only few adverse events but imatinib appeared to provide insufficient effects on progression-free and overall survival. Just one out of 23 patients with KIT expressing MCCs responded partially and the progression happened rapidly after 1 or 2 months in most of the patients. Thus, the study was prematurely discontinued [29].

Kartha and Sundram [48] examined primary and metastatic MCCs (*n* = 32) to evaluate the expression and mutations of KIT and PDGFR in MCCs. KIT expression was found in 53% of the cases. Coexpression of KIT and SCF was shown in 16% only, whereas coexpression of PDGFR-*α* and its ligand PDGF-A was found in 81% of the cases. In this study, no activating mutations could be found (KIT exons 9, 11, 13, and 17 and PDGFR-*α* exons 10, 12, and 18 were analyzed). Therefore, efficacy of imatinib is questionable, also considering results of Samlowski et al. [29].

Somatostatin receptors were also reported to be expressed in MCCs. Somatostatin analogue octreotide showed unfruitful results concerning tumor regression with a partial response rate of 3% (*n* = 58) (Table 2) [30]. Radiopeptide therapy is used to treat neuroendocrine tumors by binding of edotreotide (DOTATOC, which contains the active peptide of somatostatin, namely, octreotid) to somatostatin receptors. In one case, the radiolabeled somatostatin analog 90Y-DOTATOC led to complete remissions after a few days, but relapse and progression occurred within weeks. It has to be pointed out that the tolerability was very good (Table 2) [31]. Lanreotide, a nonradiolabeled somatostatin analog, was intramuscularly administered in one patient every two weeks and showed a complete response of the lesion. Recurrences occurred within 7 months after the first injection (Table 2) [32].

Oblimersen sodium (Genasense), which inhibits the production of Bcl-2 (which is a protein acting against apoptosis in cancer cells), was applied intravenously to 12 patients and showed no responses (Table 2) [33].

## 5. Immunotherapeutic Strategies

MCC is associated with immunosuppression [1, 4]. Cases of spontaneous regressions of MCCs were reported that were deemed to be caused by the regained activity of the immune system [56, 57]. MCCs that showed a high infiltration with CD8+ lymphocytes were attributed to a better prognosis (100% MCC-specific survival, *n* = 26) compared to MCCs with lower infiltration (60% survival, *n* = 120) [58]. 

Some patients developed MCC during treatment with tumor necrosis factor (TNF) alpha inhibitors, which usually promotes inflammatory response. Therefore, TNF-alpha inhibitors are assumed to increase the risk of occurring of MCC [59, 60]. 

The detection of the Merkel cell polyomavirus (MCV) and its genetic material, found in MCC tumor cells of about 80% of the concerned patients, provided new opportunities and possibilities regarding therapy strategies [61, 62]. For instance, interferons (*α* and *β*) have been suggested to be a possible therapeutic option [63]. However, there are only few reports on the use of immunomodulating substances in MCC. In two case reports, interferon-*α*-2b caused severe asthenia and depression, leading to discontinuation of the therapy, considering that no tumor regression was observed. The tumors were positive for MCV [63]. Recently, it has been shown that viral T-antigens represent an important signal for MCV-infected MCC cells concerning survival and growth [64]. Consequently, the inhibition of T-antigens might be a therapeutic option. 

Imiquimod, which induces immune response by binding to TLR7 (toll like receptor-7, located on the surface of immune cells, e.g., macrophages), was topically applied to MCC (*n* = 1) and combined with radiotherapy. Complete response of the lesion lasted 7 months [65].

Another new therapeutic approach is based on cytokine inducted inflammatory response. To avoid systemic reactions, fusion proteins were developed consisting of antibodies and cytokines, which bind to their corresponding antigens located on the tumor cell surface [66]. In general, combined therapy of cytokine-based antibodies and regular chemotherapy seems to be well tolerated. 

## 6. Conclusions

There is a considerable lack of prospective clinical studies and in particular randomized controlled studies that evaluated the therapeutic options for MCC. For the time being, nearly all conclusions are based on case series or even isolated cases and theoretic considerations, rather than evidence-based medicine. 

With respect to chemotherapeutics or molecular-targeted agents, no convincing responses were reported; at best the responses lasted a few months or just several weeks. There were no controlled studies. Thus, the question arises whether there truly is a benefit of chemotherapy for MCC; if any, the benefit is unlikely to be relevant. Because of frequent comorbidities of the older patient collective, it is necessary to develop therapy modalities that are effective and more likely to be tolerated. The recent insight of an association of MCC to MCV infection and immunosuppression enables new therapeutic options, for example, the inhibition of viral T-antigens, that requests further investigations. Currently, there is an ongoing phase II trial study with the purpose of placing the gene for interleukin-12 into MCC cells by intratumoral injection so that humans build up an immune defense and may kill tumor cells. Other promising options might be immune-response modifiers.

This emerging disease requires more clinical research. A first step might be the implementation of an MCC registry. More important, however, would be randomized controlled studies in this field, which are urgently needed. The often rapid deterioration of this cancer and the frequent comorbidities might require rather simple protocols. The rareness of the disease calls for the implementation of an MCC network to collect sufficient numbers of patients in an MCC registry and multicenter clinical trials.

## Figures and Tables

**Table 1:** Chemotherapeutic options: polychemotherapy and monotherapeutic options.

	Number of patients	Intent of chemotherapy	Agents used	Response	Time to progression	Survival
McAfee et al. [14] *prospective *	*n* = 34 9 patients received CTx local to metastatic disease	Adjuvant	Carboplatin, cisplatin, VP-16, etoposide	NDA	5-year local control of all cases: 94%; 5-year locoregional control of all cases: 80%	5-year survival of all cases: 37%; 5-year DSS of all cases: 52%

Poulsen et al. [15] *prospective *	*n* = 41 local or locoregional disease	Adjuvant (72%); therapeutic (28%)	CTx combined with RTx; etoposide, carboplatin	NDA	NDA	3-year OS 76%; 3-year DFS 65%

George et al. [16] *retrospective *	*n* = 1 metastatic disease	Adjuvant	Cyclophosphamide, doxorubicin, vincristin	CR: 4-5 months	4 months	NDA

Redmond III et al. [17] *retrospective *	*n* = 6 metastatic disease	Adjuvant	Cisplatin + etoposide or Cisplatin + etoposide + cyclophosphamide or Cyclophosphamide + doxorubicin + vincristin	CR in 5 patients	CR for a median of 3,5 months	median OS of 6,5 months

Azagury et al. [18] *retrospective *	*n* = 1 metastatic disease	Adjuvant	First line: VP-16 + cisplatin + doxorubicin + bleomycin — Second line: RTx combined with methotrexate + cyclophosphamide + VP-16	CR after 6 cycles — CR after 4 months	15 months until relapse — NDA	NDA

Bajetta et al. [19] *prospective *	*n* = 30 NET (1 MCC among all cases) locally advanced or metastatic disease	Adjuvant	5-Fluorouracil + dacarbazine + epirubicin	CR in 2 patients (in 1 patient with MCC); PR in 7 patients	CR of MCC for 21+ months; median duration of response of all cases was 10 months (range, 5+ to 24+)	NDA

Eng et al. [20] *retrospective *	*n* = 88; 43 patients received RT and/or combined CTx local to metastatic disease	Adjuvant	19% received CTx without RTx most common regimen: VP-16 + cisplatinum	NDA; of all cases 12% had persistent disease, 40% had recurrent disease	Of all cases median time to recurrence was 8 months	NDA

Eng et al. [21] *retrospective *	*n* = 46 9 patients received CTx metastatic disease	Adjuvant	+/− RTx most common regimen: carboplatin + etoposide + vinblastin	NDA	Overall median time to recurrence was 9 months	OS rate of all cases: 37%

Veness et al. [22] *retrospective *	*n* = 86 7 patients received CTx locoregional disease	Adjuvant	most common: based on platinum	NDA; 55% of all cases experienced a relapse	median time to relapse was 3–7 months	5-year OS 47%; DFS rate 25%

Voog et al. [23] *retrospective *	*n* = 107 advanced or metastatic disease	Adjuvant	different regimens; NDA	ORR to first line CTx: 64%	NDA	3-year OS was 17% (metastasis) and 35% (locally advanced)

Allen et al. [24] *retrospective *	*n* = 251 28 patients received CTx local or regional disease	Adjuvant	most common regimen: carboplatin + etoposide	NDA; disease recurred in 102 patients	of all cases median time to recurrence was 9 months (range, 2 to 70 months)	5-year DFS of all cases: 48%; 5-year DSS of patients receiving CTx: 28%

Wobser et al. [25] *retrospective *	*n* = 5 metastatic disease	Adjuvant	+RTx liposomal doxorubicin	PR in 4 patients	an average of 2 months until progression	Survival time: range 3 to 20 months

Schlaak et al. [26] *retrospective *	*n* = 4 metastatic disease	Adjuvant	Etoposide	CR in 3 patients	2 of CR lasted for 16 and 36 months	NDA

Tai et al. [27] *retrospective *	*n* = 204 locoregional and metastatic disease	Adjuvant	most common regimens: Cyclophosphamide + doxorubicin (or epirubicin) + vincristin +/− prednisone — Etoposide + cisplatin (or carboplatin)	ORR 75,7% (35,1% CR, 35,1% PR, 5,4% minor responses) — ORR 60% (36% CR, 24% PR)	Median response duration of patients receiving CTx: 1–12 months	Median OS of all cases: 21,5 months (range, 1 to 118 months); 2-year OS of all cases: 36%; 5-year OS of all cases: 17%

NDA: no data available; NET: neuroendocrine tumor(s); MCC: merkel cell carcinoma; PR: partial response; CR: complete response; CTx: chemotherapy; RTx: radiation therapy; Sx: surgery; DFS: disease free survival; OS: overall survival; ORR: overall response rate; DSS: disease-specific survival.

**Table 2:** Molecular-targeted agents.

	Number of patients	Agent used	Dosage	Tolerability	Response	Median time to progression
Davids et al. [28]	*n* = 1 metastatic disease	Pazopanib	800 mg daily	Minimal adverse effects; dose reduction after gallstone pancreatitis to 400mg daily	After 2 months: CR of primary lesion, PR of metastatic lesions	4 months

Samlowski et al. [29]	*n* = 23 metastatic or unresectable disease	Imatinib	400 mg daily	Mainly Grade 1-2 toxicities; 3 episodes of Grade 4 toxicities; Grade 3 toxicities in 3 patients	no CR; PR: 4%	1-2 months

Di Bartolomeo et al. [30]	*n* = 58 metastatic disease (neuroendocrine tumors)	Octreotide	500 or 1000 micrograms 3 times a day	Carcinoid syndrome and abnormal urinary 5-hydroxy-indoloacetic acid excretion were reported	Median survival time of 22 months (range, 1–32+ months); PR: 3%	Disease stabilized for at least 6 months (range, 1–32+ months)

Meier et al. [31]	*n* = 1 locoregional disease	90Y-DOTATOC; targeted radiotherapy	85 mCi	Fatigue was main side effect	PR after 1 week; CR after 4 weeks	10 weeks until locoregional relapse

Fakiha et al. [32]	*n* = 1 clinically locoregional disease	Lanreotide	15 mg i.m. injection every two weeks	No side effects reported	Clinically CR after 2 months	7 months until relapse

Shah et al. [33]	*n* = 12 metastatic or regionally recurrent disease	Oblimersen sodium	7 mg per kilogram daily	Including Grade 3 and Grade 4 events, for example, lymphopenia, renal failure, cytopenia, and hyperkalemia	No responses; stable disease in 3 patients	PD in 9 patients

CR: complete response; PR: partial response; PD: progressive disease.
